# Cardiac Transcriptomics Reveals That MAPK Pathway Plays an Important Role in Hypoxia Tolerance in Bighead Carp (*Hypophthalmichthys nobilis*)

**DOI:** 10.3390/ani10091483

**Published:** 2020-08-24

**Authors:** Ying Zhou, Weiwei Luo, Xiaomu Yu, Junru Wang, Yizhao Feng, Jingou Tong

**Affiliations:** 1 State Key Laboratory of Freshwater Ecology and Biotechnology, Institute of Hydrobiology, Innovation Academy of Seed Design, Chinese Academy of Sciences, Wuhan 430072, China; Memoria_Y@163.com (Y.Z.); weiweiluo66@163.com (W.L.); jrwang1021@163.com (J.W.); yzfenguse@163.com (Y.F.); 2 University of Chinese Academy of Sciences, Beijing 100039, China

**Keywords:** bighead carp, hypoxia stress, RNA-seq, cDNA-AFLP, MAPK signaling pathway

## Abstract

**Simple Summary:**

Acute hypoxia treatment was performed in juvenile bighead carp (*Hypophthalmicthys nobilis*) by decreasing water O_2_. The results showed that blood lactate and serum glucose increased significantly under hypoxia stress, and some differentially expressed genes were identified among hypoxia tolerant, hypoxia sensitive, and normoxia control groups. Differentially expressed genes between hypoxia tolerant and hypoxia sensitive groups were mainly involved in mitogen-activated protein kinase (MAPK) signaling, insulin signaling, apoptosis, tight junction, and adrenergic signaling in cardiomyocytes pathways, of which MAPK signaling pathway played a key role in cardiac tolerance to hypoxia in bighead carp. These results provide a basis for understanding the physiological and molecular mechanisms underlying hypoxia response in fish and a guide for future genetic breeding programs for hypoxia resistance in bighead carp.

**Abstract:**

As aquatic animals, fishes often encounter various situations of low oxygen, and they have evolved the ability to respond to hypoxia stress. Studies of physiological and molecular responses to hypoxia stress are essential to clarify genetic mechanisms underlying hypoxia tolerance in fish. In this study, we performed acute hypoxia treatment in juvenile bighead carp (*Hypophthalmicthys nobilis*) by decreasing water O_2_ from 6.5 mg/L to 0.5 mg/L in three hours. This hypoxia stress resulted in a significant increase in blood lactate and serum glucose. Comparisons of heart transcriptome among hypoxia tolerant (HT), hypoxia sensitive (HS), and normoxia control (NC) groups showed that 820, 273, and 301 differentially expressed genes (DEGs) were identified in HS vs. HT, NC vs. HS, and NC vs. HT (false discovery rate (FDR) < 0.01, Fold Change> 2), respectively. KEGG pathway enrichment showed that DEGs between HS and HT groups were mainly involved in mitogen-activated protein kinase (MAPK) signaling, insulin signaling, apoptosis, tight junction and adrenergic signaling in cardiomyocytes pathways, and DEGs in MAPK signaling pathway played a key role in cardiac tolerance to hypoxia. Combined with the results of our previous cDNA-amplified fragment length polymorphism (cDNA-AFLP) analysis of hypoxia stress in this species, such genes as *stbp2*, *ttn*, *mapk*, *kcnh*, and *tnfrsf* were identified in both studies, representing the significance of these DEGs in hypoxia tolerance in bighead carp. These results provide insights into the understanding of genetic modulations for fish heart coping with hypoxia stress and generate basic resources for future breeding studies of hypoxia resistance in bighead carp.

## 1. Introduction

Aquatic animals such as fish are frequently exposed to hypoxic environments owing to low atmospheric pressure and eutrophication in water [1,2]. Hypoxic conditions have been known as a serious problem in aquaculture systems [3]. Hypoxia stress limits the transport of dissolved oxygen (DO) across the gill membrane and the level of cellular metabolic activity, which inhibits vital activities of the whole organism in turn, including feeding and growth [4]. In fish, hypoxic death is relevant to catastrophic loss of substrate, failure of vital adenosine triphosphate (ATP) consuming processes, accumulation of toxic levels of waste products (protons/lactate), and cellular necrosis [5]. Many responses for fish to adopt different mechanisms to tolerate hypoxia are behavioral, including surface breathing, reduced activity, or increased ventilation rate [6]. Fish also exhibit adaptive responses to hypoxia, including discontinuation of processes requiring substantial energy output, such as cell growth/proliferation, protein synthesis, and locomotion [7,8]. Different fish species vary greatly in their ability to tolerate and survive when the content of oxygen in water decreases below a certain critical value [9]. Thus, unearthing the molecular mechanisms of hypoxia adaptation and tolerance in fishes will not only help us to understand evolution of the hypoxia-related signaling pathway but also guide us in the breeding of hypoxia-tolerant fish strains [10].

In vertebrates, the precise establishment and regulation of systems, such as heart, erythrocytes, and vasculature, provide a major basis for O_2_ homeostasis [11]. Insufficient blood supply leads to tissue hypoxia in the heart during acute infarction and chronic ischemia [12]. The energy of myocardial metabolism is almost provided by aerobic metabolism, which is very sensitive to ischemia and hypoxia, and hypoxia is the main factor of myocardial ischemia [13]. Under mild or moderate hypoxia conditions to alleviate hypoxia, the heart can be well adapted to the hypoxic environment through organic regulation of the whole body [14]. The severity of injury depends on the intensity of hypoxic stimulation and relates to the tolerance of heart to hypoxia as well [15]. In mammals, many factors, such as ATP-sensitive potassium (KATP) channels, reactive oxygen species (ROS), and mitogen-activated protein kinase (MAPK) pathways are involved in the mechanism of increased tolerance of chronically hypoxic hearts [16]. It has been revealed that general transcriptional changes are related to metabolic depression, energy conservation, protein synthesis, cell growth, proliferation and muscle contractility [17]. However, in fish species, knowledge for genetic variations of individual fish responsible to hypoxia stress within a population is limited so far.

Hypoxia is one of the most serious threats in aquaculture of many economic fishes. Bighead carp (*Hypophthalmichthys nobilis*) is one of the most important aquaculture fish species in China and some Asian countries. Together with silver carp (*Hypophthalmichthys molitrix*), bighead carp has also been introduced to many countries for aquaculture production as food fish and biological control of plankton in a variety of water systems, and it has been intensively cultured since the 1960s [18]. Compared with other fish species, the asphyxiation point of bighead carp (0.68–0.34 mg/L) is a little lower than silver carp (0.72–0.34 mg/L) and higher than grass carp (0.51–0.3 mg/L), common carp (0.34–0.3 mg/L), and crucian carp (0.13–0.11 mg/L) [19]. As a relatively hypoxia-sensitive species, bighead carp, together with silver carp, Eurasian perch (*Perca fluviatilis*), and Wuchang bream (*Megalobrama amblycephala*), have suffered serious losses because of hypoxia stress due to weather changes in pond culture every year. Thus, it is desirable to determine the molecular mechanisms of hypoxia and improve hypoxia tolerance capabilities of bighead carp. However, few studies have been reported about the molecular and cellular adaptations to hypoxia in bighead carp. To date, some studies have reported the results of transcriptome analyses in response to hypoxia in fishes, such as viviparous (*Xiphophorus*) and oviparous (*Oryzias*) fishes [20], threespine stickleback (*Gasterosteus aculeatus*) [21], Atlantic salmon (*Salmo salar*) [22], and blunt snout bream (*Megalobrama amblycephala*) [23]. Most of these studies focus on transcriptome analyses in liver or muscle tissues and their responses to acute or chronic hypoxia stress compared with normoxia samples. Transcriptomic studies of the heart have been used to investigate gene expression changes in response to hypoxia stress only in tilapia [17] and Schizothoracine fish (*Gymnocypris eckloni*) [24].

We hypothesized that the genetic architecture of hypoxia responses may, to some extent, be differently determined and/or regulated within the bighead carp population. Here, we performed comparative transcriptome analyses of sensitive and tolerant fish under acute hypoxia stress using heart tissues and combined with the results of our previous cDNA-AFLP analysis of sensitive and tolerant bighead carp under the same treatment. We aimed to find novel candidate genes and biological pathways that may be associated with the tolerance of bighead carp to hypoxia stress, which would provide a better understanding of adaptive and genetic mechanisms in response to hypoxia stress in fish.

## 2. Materials and Methods

### 2.1. Experimental Fish and Hypoxia Treatment

One hundred and fifty healthy bighead carp individuals at the age of six months of similar size (mean weight: 132.20 ± 5.66 g) were sampled from the Zhangdu Lake Fish Farm (Wuhan, China). Fish were temporally maintained in a tank (water depth: 70 cm, volume: 500 L) with a recirculating freshwater system for a week (water temperature 27 ± 1 °C and dissolved oxygen (DO) > 6 mg O_2_/L. The fish were fed with pellet feeds twice a day and stopped feeding the night before hypoxia treatment started. The photoperiod was adjusted to 12D:12L in the room. During hypoxia treatment, DO was reduced from 6.0 ± 0.1 to 0.5 ± 0.1 mg/L by pumping nitrogen into the water through a nitrogen gas cylinder over 1 h, and then maintained at 0.5 ± 0.1 mg/L for 2 h. In order to eliminate possible errors caused by environmental factors, the experiment was done in one day and all experiment fish were subjected to hypoxia stress in one batch. DO values were monitored continuously in this study using an YSI Model 580 dissolved oxygen meter (Geo Scientific Inc., Yellow Springs, OH, USA). After 50 min for hypoxia stress, some fish started to lose balance, and the first batch of five fish to lose their balance were immediately removed from the tank and sampled as the hypoxia-sensitive (HS) group. With the extension of hypoxia duration, more and more fish lost their balance and were continuously removed from the tank, and last five fish left, which were still swimming normally in the tank, were regarded as the hypoxia-tolerant (HT) group. The detailed information for the decreasing of DO and the number of loss-balanced fish were recorded and showed in Figure S1. The fish in the normoxia control group (NC) were kept within normal oxygen saturation for the same time period.

After the experiment, blood (five replicates of each group) was collected from a caudal puncture in an EDTA-treated tube and then transferred to microfuge tubes. The heart tissues from HS, HT, and NC groups with three replicates were quickly sampled and stored in liquid nitrogen until RNA isolation. All experiments involving fish in this study were conducted in strict accordance with the recommendations in the Guide for the Care and Use of Laboratory Animals of the Institute of Hydrobiology, Wuhan, China (20181205-2).

### 2.2. Physiological and Biochemical Responses of Bighead Carp to Hypoxia

Hb was determined from whole blood, while lactate and glucose were measured from the serum samples. Hb was measured using a Hemoglobin Test Solution Kit (Njjcbio, Nanjing, China). Serum glucose was determined by Glucose Assay Kit (Njjcbio, Nanjing, China) based on an enzymatic method which uses glucose oxidase and peroxidase reaction to form a red quinoneimine dye absorbing at 505 nm. Quantitative determination of lactate was done using Lactic Acid Assay Kit (Njjcbio, Nanjing, China) by reducing NBT to a purple compound that absorbs 530 nm.

### 2.3. RNA Extraction and Transcriptome Sequencing

Total RNA was extracted using TRIzol Reagent (Invitrogen, Carlsbad, CA, USA) according to the manufacturer’s instruction. The quality of RNA was assessed by running electrophoresis on 1.5% agarose gel and a NanoDrop 2000 Spectrophotometer (Thermo Scientific, Wilmington, DE, USA), respectively. Illumina cDNA libraries were constructed according to the manufacturer’s recommendation (NEB, Ipswich, MA, USA) and then sequenced using Illumina Novaseq for 2 × 150 bp pair-end (PE) sequencing at the Biomarker Technologies Corporation (Beijing, China). First, raw reads of Fastq format were processed through in-house Perl scripts, and all reads with sequencing adapters and nucleotides in reads with a quality value <20 in both ends were removed. High-quality sequences of clean reads were obtained by eliminating reads with adapters, ploy-N, and low-quality reads from raw data. Then, Q20, Q30, GC-content, and sequence duplication level of the clean data were also calculated.

### 2.4. Transcriptome Alignment and Functional Annotation

We analyzed the quality of data filtering using FastQC. The clean data from nine transcriptome libraries were assembled together by Trinity software to get unigenes that were more accurate and comprehensive for samples from same species without genome-wide reference. Then, unigenes were generated by connecting the contigs longer than 200 bases to obtain sequences that could not be extended on either end, and maximum length non-redundant unigenes were acquired by further splicing and assembling by TGICL clustering software (J. Craig Venter Institute, Rockville, MD, USA). After that, we annotated the gene fragments against the National Center of Biotechnology Information (NCBI) Nr protein database, The database of Clusters of Protien homology (KOG) database, Swiss–Prot database, Kyoto Encyclopedia of Genes and Genomes (KEGG) database, and Pfam database. Gene Ontology (GO) annotations of unigenes were performed using Blast2GO based on results of the Nr database annotation. These unigenes were aligned with the Nr database to search for proteins with the highest sequence similarity to the given unigenes and annotate their protein functions using the Blastn tool.

### 2.5. Analysis of Differential Gene Expression

Differential expression analyses for three groups of fish were performed using the DESeq R package applying the MA-plot-based method with the random sampling model (MARS). Differentially expressed genes (DEGs) with significant expression abundance among groups were selected using the parameters |log2 (fold change)| > 1 and false discovery rate (FDR) ≤ 0.01. GO and KEGG pathway analyses were performed based on the DEGs.

cDNA-AFLP analysis of the DEGs related to hypoxia stress in bighead carp was also performed in our lab. In order to verify the accuracy and significance of the DEGs identified in the present study, those DESs were compared with candidate genes from the cDNA-AFLP analysis in our previous report [25].

### 2.6. Validation of RNA-Seq Results by qRT-PCR

RNA samples with three biological replicates were used for quantative real-time PCR (qRT-PCR) to validate the results of transcriptome data. The cDNA was synthesized from 1000 ng of total RNA for each sample using Prime-Script^TM^ RT Reagent Kit (TaKaRa, Dalian, China). Nine pairs of primers were designed by Primer 5.0 software. The qPCR amplification was performed in a total reaction volume of 6.5 μL Power SYBR Green PCR Master Mix (Applied Biosystems, Foster City, CA, USA), 0.2 μM of each forward and reverse primer, 1.2 μL diluted cDNA, and 4.5 μL sterile distilled water, and then was run on the StepOneTM Real-Time PCR System (Applied Biosystems, Foster City, CA, USA). All the samples were analyzed in triplicate, and the fold changes of gene expression were calculated using 2^−ΔΔCT^ method.

## 3. Results

### 3.1. Changes of Blood Parameters after Hypoxia Stress

**Figure 1:**
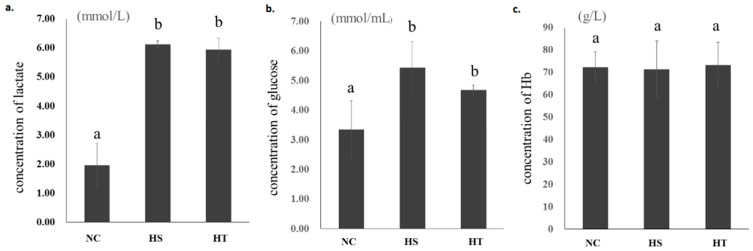
Glucose (**a**), lactic acid (**b**), and hemoglobin (Hb) (**c**) concentrations in blood samples of the normoxia control (NC), hypoxaia sensitive (HS), and hypoxia tolerant (HT) groups of bighead carp. ^a,b^ Means with different letters differ significantly between control and hypoxia stress groups.

Glucose and lactic acid were found to be significantly increased under lower oxygen concentration, but there was no difference between hypoxia sensitive (HS) and hypoxia tolerant (HT) groups (Figure 1). Hemoglobin (Hb) was expected to increase as oxygen concentration decreased; however, there were no significant treatment effects seen about Hb between control and hypoxia stress groups (Figure 1).

### 3.2. Raw Sequencing Data and De Novo Transcriptome Assembly

After quality filtering, the RNA-seq of nine heart samples yielded around 69.37 Gb high-quality clean data, with a mean clean bases of 6.28 Gb. The Q30 values of each sample were up to 93.96%, and GC-content of each sample ranged from 44.72% to 46.91% (Table S1). The clean reads obtained from nine transcriptome libraries were assembled to full-length transcripts, and a total of 100,817 unigenes were achieved after elimination of redundant transcripts. The N50 values of the transcripts and unigenes obtained were 2675 bp and 1633 bp, respectively. Summary data of the assembled transcripts and unigenes were shown in Table S2, and the number of unigenes per size, which normally decreases continuously, is given in Table S2 and Figure S2.

### 3.3. Functional Annotation and Classification of Unigenes

In order to obtain comprehensive functional information, all unigene sequences were subjected to a search against Nr, Swissprot, KOG, and KEGG database. We obtained annotations as follows: 25,295 in Nr, 13,208 in Swissprot, 14,613 in KOG and 12,071 in KEGG (Table 1), respectively.

GO assignment was performed to classify functions of the predicted bighead carp genes (Table 1 and Figure S3). Based on sequence homology, a total of 13,996 unigenes were annotated to 58 terms of GO classification, which were classified into three major functional categories, including 23 GO Slim Terms of Biological Process category, 19 GO Slim Terms of Cellular Component category, and 16 GO Slim Terms in Molecular Function category, respectively.

### 3.4. Differential Expression Analysis and Functional Annotation

**Figure 2:**
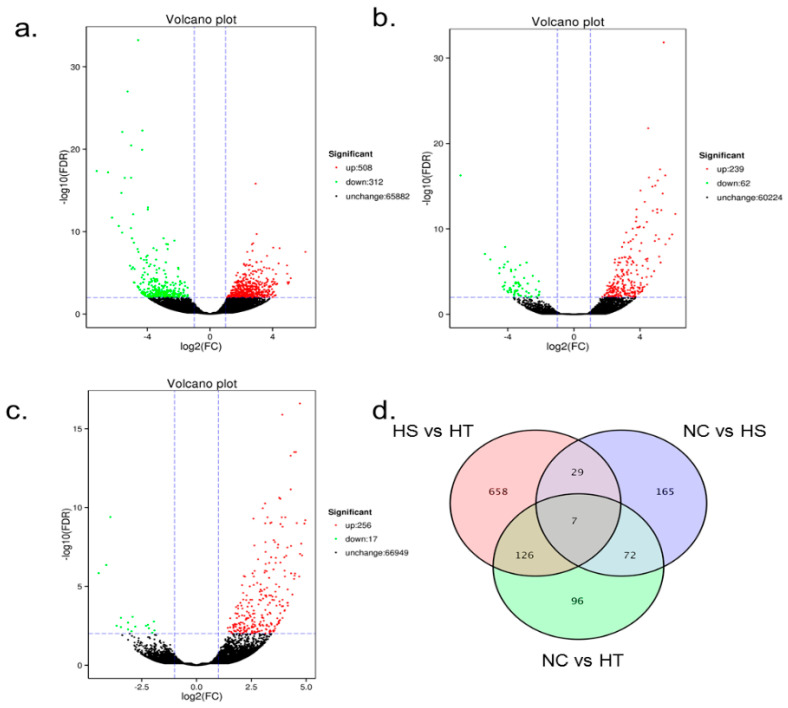
Patterns of unigenes differentially expressed between HS vs. HT (**a**), NC vs. HT (**b**) and NC vs. HS (**c**). Red or green dots represent differentially expressed unigenes (DEGs), black dots represent non differentially expressed unigenes. (**d**) Venn diagrams describe overlaping DEGs among three groups of samples in heart transcriptiome of bighead carp under hypoxia stress.

In total, the number of DEGs in bighead carp exposed to hypoxia were as follows: 820 DEGs between HS and HT groups (508 up- and 312 down-regulated in HT group), 273 DEGs between NC and HS groups (256 up- and 17 down-regulated in HS group), and 301 DEGs between NC and HT groups (239 up- and 62 down-regulated in HT group) (Figure 2).

**Figure 3:**
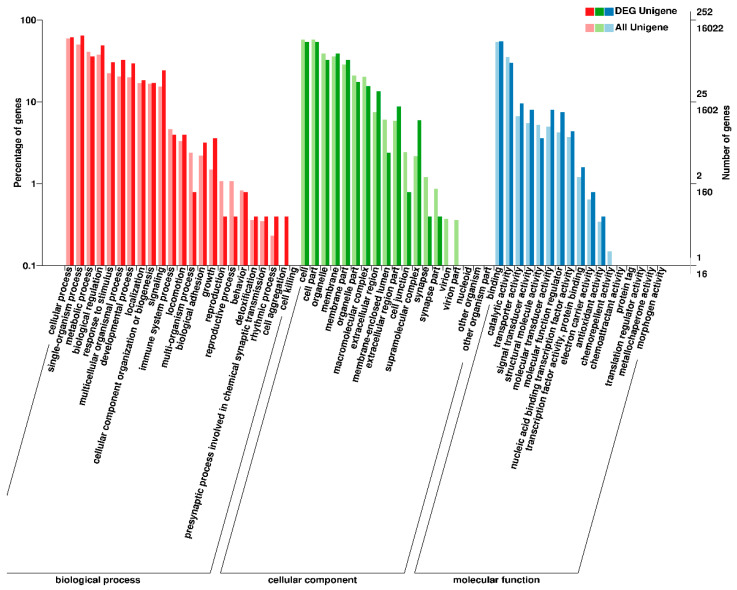
Gene Ontology (GO) terms associated with DEGs between HS and HT groups, including three main categories: biological processes, cellular component, and molecular function.

To elucidate biological events of the DEGs from heart tissues, which were mainly involved in response to hypoxia in bighead carp, GO term enrichment analyses were conducted. The GO functional enrichment analysis showed that a significant percentage of genes were clustered into locomotion (GO: 0040011), rhythmic process (GO: 00048511) and chemoattractant activity (GO: 0042056) in NC vs. HS (Figure S4); transcription factor activity (GO: 0003700), electron carrier activity (GO: 0009055), rhythmic process (GO: 00048511), and molecular transducer activity (GO: 0060089) in NC vs. HT (Figure S5). Additionally, some biological processes including extracellular region (GO: 0005576), supramolecular complex (GO: 0099080), and growth (GO: 0040007) were significantly clustered in HS vs. HT (Figure 3).

**Figure 4:**
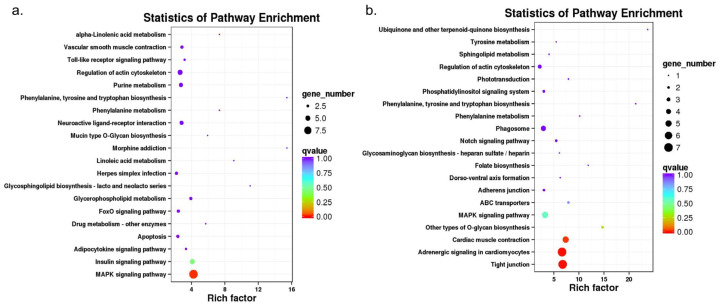
Kyoto Encyclopedia of Genes and Genomes (KEGG) pathway enrichment of the DEGs in up-regulation (**a**) and down-regulation (**b**) of genetic responses to hypoxia stress in bighead carp. The size of colored dots represents the number of significant DEGs associated with each corresponding pathway.

The KEGG analysis revealed that the FoxO signaling pathway, arginine biosynthesis, adipocytokine signaling pathway, MAPK signaling pathway, and mTOR signaling pathway were significantly enriched in both NC vs. HT and NC vs. HS (Figures S6 and S7). Up-regulated DEGs of HS vs. HT were mainly classified into MAPK signaling pathway, insulin signaling pathway, adipocytokine signaling pathway, and apoptosis, while down-regulated DEGs were classified into cardiac muscle contraction, tight junction, adrenergic signal in cardiomyocytes, and other types of O-glycan biosynthesis pathways (Figure 4).

### 3.5. Critical DEGs Involved in the Response to Hypoxia Stress

**Figure 5:**
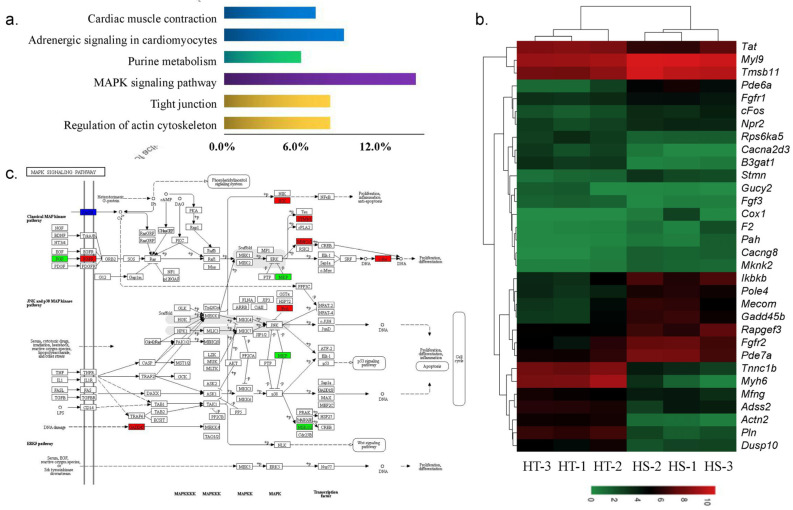
Patterns of selected DEGs between HS and HT groups: (**a**) main KEGG pathways classified by the DEGs in HS vs. HT; (**b**) heat-map of selected significant genes from main pathways; (**c**) annotation diagram of MAPK signaling pathway and its related DEGs.

To identify a variety of adaptive responses to hypoxia in bighead carp, which have more value in aquaculture, we focus on the DEGs in HS vs. HT groups. Ten key pathways and 33 key DEGs associated with hypoxia tolerance were identified from top KEGG classified significantly (Table 2 and Figure 5). These key pathways were mainly involved in environmental information processing, cellular processes, and organismal system, such as the MAPK signaling pathway, insulin signaling pathway, and regulation of actin cytoskeleton enriched by up-regulated DEGs, while tight junction, adrenergic signaling in cardiomyocytes, and cardiac muscle contraction down-regulated DEGs. Compared with the DEGs from hypoxia sensitive group, out of thirty-three candidate genes were 22 up-regulated and eleven down-regulated in hypoxia tolerant group, respectively.

To verify the accuracy and significance of DEGs identified in the present study, we also combined with the genes previously obtained from cDNA-AFLP analysis of heart for hypoxia tolerant and sensitive bighead carp [25]. The results showed that two shared genes were identified in both transcriptome and cDNA-AFLP analyses, including saxitoxin and tetrodotoxin-binding protein 2 (*Stbp2*) and *Titin* (Table 3). Six genes from similar gene families (Mapk, Tnfrsf and Kcnh) were also identified when compared with two studies of hypoxia treatment in bighead carp. *Mapk6*, *Kcnh1*, and *Tnfrsf14* showed different expressions in cDNA-AFLP analysis [25], while *Map3k15*, *Kcnh2*, and *Tnfrsf12* had significantly different expressions only in transcriptome analysis in this study.

### 3.6. Validation of RNA-Seq Results by qPCR

To validate the accuracy of RNA-seq data obtained in this study, qRT-PCR were performed for randomly selected 9 DEGs: growth arrest and DNA damage inducible beta (*Gadd45b*), Krueppel-like factor 9 (*Klf9*), cysteine/serine-rich nuclear protein 1 (*Csrnp*), forkhead box protein G1 (*Foxg1*), CCAAT/enhancer-binding protein delta (*Cebpd*), smoothelin (*Smtn*), *Ttn*, fibroblast growth factor 3 (*Fgf3*), and MAP kinase-interacting serine/threonine-protein kinase 2 (*Mknk2*). Results from qRT-PCR were compared to the data obtained by RNA-seq. The expression patterns for the nine DEGs were similar using two methods, which confirmed that the data obtained from RNA-seq were reliable (Figure S7).

## 4. Discussion

Aquatic environments exhibit wider spatial and temporal variations in their oxygen content compared to terrestrial environments [26]. Thus, fish and other aquatic animals are frequently exposed to various oxygen levels. Hypoxia is one of significant environmental stresses that have a marked impact on the survival, growth, and development of aquatic organisms [24,27]. Previously, oxyconforming responses to hypoxia have been reported in fishes including *Galaxias maculatus* [28], *Acipenser naccarii* [29] sea worm *Sipunculus nudus* [30] and estuarine fish *Leiostomus xanthurus* [31]. As a relatively hypoxia-sensitive freshwater species, bighead carp is easy to be affected by the decrease of dissolved oxygen due to weather changes or eutrophication in the process of growth [32]. Growth (body size, weight, age) and health state are both correlated with the tolerance of anoxia. Thus, to measure the hypoxia tolerance of bighead carp more accurately, fish samples selected in this study were from the same pond with similar growth background. The loss of equilibrium (LOE) represents partial oxygen pressure at which fish can maintain balance, which is an indicator for measurement of hypoxia tolerance [33]. In this study, LOE was chosen as the indicator of hypoxia, and the DO suffocation point of bighead carp was 0.5 mg/L, which was consistent with 0.5 mg/L of blunt snout bream [34].

Under hypoxia stress, fish can slow down their metabolic rate to match reduced energy supply [1,35]. In general, animals delay the depletion of glycogen stores and the accumulation of toxic levels of lactate by reducing their metabolic rate. Lactate is a kind of physiological end-product produced by pyruvate reduction during glycolysis [36]. In the present study in bighead carp, a significant increase of blood lactate in hypoxic conditions indicates that alteration of lactate metabolism may be one of the prominent components of the metabolic stress responses [36]. Simultaneously at this stage, serum glucose at experimental hypoxia level also significantly increased when compared to normoxic conditions. As oxygen concentration decreased, Hb was expected to increase for the improvement of the transport capacity of oxygen [37,38], however, there were no significant treatment effects seen about Hb between normoxic control group and hypoxia groups. In the study of Indian catfish, Hb didn’t change in an hour under hypoxia as well but showed a decreasing trend after 2 h [38]. Haematocrit value may be a meaningful index, which wasn’t taken in this study, and we would consider taking haematocrit value as an index of possible changes of red cell numbers or volumes in future genetic and transcriptome/proteomics studies for acute hypoxia in bighead carp or other fish species.

To date, several oxygen sensors have been reported to regulate hypoxia adaptation in fishes [26]. As we all known, vertebrates adapt to low oxygen conditions through a conserved transcriptional response pathway mediated by the key transcription factor, hypoxia-inducible factor (HIF), which has been well characterized in mammals [39]. In animals, HIF-1, composed of HIF-1α and HIF-1β, is able to control a variety of cellular and systemic homeostatic responses to hypoxic stress by the oxygen dependence of HIF protein level [4,35]. Hypoxia causes a stabilization of Hif-α protein and the formation of the active Hif-α/Hif-1β dimer, but the transcription of hif genes remains unaltered, therefore mRNA profiling of hif isoforms does not provide any indication for the activation of Hif signaling in most mammals [40]. In fish, analysis of hif mRNA concentration indeed provides evidence for hypoxia-activated Hif signaling, which is different from that of mammals. Acute hypoxia can induce *Hif1* expression in brain and liver, and chronic hypoxia can lead to an important change in *Hif1*a expression in fish muscle [32]. The expression of *Hif3* was significantly upregulated both in HS and HT compared with NC, but there were no obvious different expressions between HS and HT in our study. It was suggested that the hif-1α/2α expression is probably related to hypoxia treatment time, tissues and species [41]. Hif can regulate transcription of other genes involved in cellular and systemic responses to hypoxia, such as breathing, anaerobic metabolism, vasodilation, and angiogenesis [42]. However, the role of Hif-3α is less clear, and it is subject to extensive alternative splicing, which may act as inhibitor for Hif-1α and Hif-2α [43]. In Tibetan sheep, it was reported that HIF-3α may play an important role in heart responding to hypoxia to prevent damage [44]. Accordingly, besides HIF-1 signaling pathway, many other genes/pathways may also play significant roles adaptive to hypoxia, such as the MAPK signaling pathway, VEGE signaling pathway, and mTOR signaling pathway [24].

In fish, hypoxia tolerance is one of the most important traits, and it is necessary to study molecular mechanisms of hypoxia adaption before we could improve the ability of fish to tolerate hypoxia [34]. Since hypoxia is very acute in the present study, only transcriptional changes were investigated, which are immediate and fast enough to respond reliably. Although many functional or physiological changes occur at protein level, we focus on potential gene expression changes of these adaptations. Here, several pathways related to MAPK signaling pathway, insulin signaling pathway, apoptosis and FoxO signaling pathway were upregulated in the heart. While pathways related to tight junction, adrenergic signaling in cardiomyocytes, ABC transporters and phagosome were downregulated in the hearts of the hypoxia-tolerant fish, when compared to the hearts of the hypoxia-sensitive fish. It was suggested that hypoxia can slow down the processes of cell division and metabolism to decrease the energy expenditure. These results were consistent with the expression patterns of these pathways in other fishes [24,34], which would benefit to adapt to low oxygen environment for fish. Our study is completely transcriptional, and these results of gene expression changes from hypoxia-sensitive and tolerant groups may or may not reflect changes in protein level because information from transcriptome and proteome may be similar in some cases but may not be so in others.

G6pc, strongly expressed in HT group, could catalyze the dephosphorylation of glucose-6-phosphate to glucose, which is then recycled to other tissues to promote glycolysis [14]. Additionally, myosin heavy chain 6 (*Myh6*), and myosin heavy chain 7 (*Myh7*) were downregulated in HS vs. HT, suggesting that skeletal muscle may transform from fast muscle to slow muscle, thus reducing skeletal muscle contraction during hypoxia in bighead carp. It is also regarded as an adaptation strategy for fish to conserve energy by reducing swimming activity in hypoxia environments [34].

cDNA-AFLP analysis is a differential display technique, which can reduce false positives using restriction enzymes to generate cDNA-specific tags [45]. *Titin* was identified in both cDNA-AFLP [25] and transcriptome analyses in this study, confirming that it can be influenced after acute hypoxia in bighead carp, which has been reported in mammals [46,47]. *Titin* is a direct target gene of HIF1 in vivo, and hypoxia signaling controls cardiac development through HIF1 mediated transcriptional regulation of crucial components of myofibrillogenesis and the cardiac transcription factor network [46]. Although tumor necrosis factor 2 has an oxygen-dependent nature, the role of *Tnfrsfs* remains uncertain in response to hypoxia stress, although they were always induced by hypoxia [47].

MAPK is a family of enzymes involved in oxygen sensing. Previous studies have shown that hypoxia activates the MAPK signaling pathway to modulate HIF-1α activity in heart [48] and MAPK signaling pathway plays an important role in the mechanism of increased tolerance of chronically hypoxic hearts [49]. Consistent with previous studies, we observed that hypoxia led to changes in MAPK expressions in bighead carp heart [10]. In hypoxia stress environment, rat nucleus pulposus cell activates mitogen-activated protein kinase or extracellular signal-regulated kinase (MAPK/ERK) signaling pathways [50]. In down-regulated genes, *Fgf3*, one of fibroblast growth factor family members, was reported to be involved in hypoxia [51]. In the up-regulated DEGs, *Cacng8*, *Gadd45*, *cFos*, *Mknk2*, *Stmn*, *Ikbkb*, *Fgfr2*, *Mecom*, and *Fgfr1* were significantly enriched in HS vs. HT in this study (Table 2 and Figure 5). In mammals, many studies have shown an increased expression of c-Fos by hypoxia, e.g., hypoxia induces c-Fos expression in the LV and RV in rats [52] and the same held true in tissue culture cells [53]. Kim et al. [54] reported that Gadd45 is a mediator of cardiomyocyte apoptosis induced by ischaemia/hypoxia, which will increase during hypoxia. MAPKKK and MTK1 activated by GADD45-like genes can mediate activation of both p38 and JNK in response to environment stresses in human [55]. Here, the gene expression changes and MAPK signaling pathways observed in this study of transcriptional response to hypoxia stress, indicate that these above mentioned DEGs and pathways play important roles in the mechanism underlying increased tolerance of acute hypoxic hearts in bighead carp. Some of those potential genes can be used as candidate gene markers of hypoxia tolerance for possible breeding programs in the future once their functions are verified via various methods of functional genomics e.g., gene editing.

## 5. Conclusions

In summary, blood lactate and serum glucose significantly changed when bighead carp were subjected to hypoxia stress. Thirty-three key DEGs in response to hypoxic tolerance and sensitiveness were identified by transcriptome analysis and were mainly assigned to MAPK signaling pathway, suggesting that this pathway may play an important role in cardiac tolerance to hypoxia stress in bighead carp. Two candidate genes, *Stbp2* and *Titin*, were proposedly involved in hypoxia tolerance as supported by both comparative transcriptomics in this study and our previous cDNA-AFLP analyses. These findings provide a basis for understanding physiological and molecular mechanisms underlying hypoxia response in fish and a guide for future genetic breeding programs for hypoxia-resistance in bighead carp.

## Figures and Tables

**Table 1:** Annotation of the assembled gene fragments of bighead carp.

Category	Count
KOG classified sequences	14,613
Swiss-Prot	13,208
GO classified sequences	13,996
KEGG classified sequences	12,071
Nr annotations	25,295
All annotations	25,892

**Table 2:** Top KEGG pathways and DEGs in heart transcriptome between hypoxia-sensitive and tolerant bighead carp.

Regulation	Name of Pathway	Pathway ID	Genes
up	MAPK signaling pathway	ko04010	*Cacng8*, *Gadd45*, *cFos*, *Mknk2*, *Stmn*, *Ikbkb*, *Fgfr2*, *Mecom*, *Fgfr1a*
	Insulin signaling pathway	ko04910	*Cbl*, *Mknk2*, *Flot1*, *Ikbkb*, *G6pc*
	Regulation of actin cytoskeleton	ko04810	*Tmsb11*, *F2*, *Myl9*, *Fgfr2*, *Fgfr1a*
	Neuroactive ligand-receptor interaction	ko01230	*Adra2a*, *Htr1a*, *F2*, *Plg*
	Purine metabolism	ko01200	*Pde6a*, *Pole4*, *Npr2*, *Pde7a*
down	Tight junction	ko04530	*Actn2*, *Myh7*, *Myh6*
	Adrenergic signaling in cardiomyocytes	ko04261	*Tnnc1b*, *Pln*, *Rps6ka5*, *Myh7*, *Cacna2d3*
	Cardiac muscle contraction	ko04260	*Tnnc1b*, *Myh7*, *Cacna2d3*
	Other types of O-glycan biosynthesis	ko00514	*B3gat1*, *Mfng*
	MAPK signaling pathway	ko04010	*Fgf3*, *Dusp10*, *Rps6ka5*, *Cacna2d3*

**Table 3:** Hypoxia-responded genes identified by cDNA-amplified fragment length polymorphism (cDNA-AFLP) [25] and RNA-seq (this study).

RNA-Seq	cDNA-AFLP	Regulation	Groups
*Stbp2*	*Stbp2*	up	HS vs. HT
*Titin*	*Titin*	down	HS vs. HT
*Map3k15*	*-*	up	NC vs. HS
*-*	*Mapk6*	up	HS vs. HT
*Kcnh2*	*-*	down	HS vs. HT
*-*	*Kcnh1*	down	HS vs. HT
*Tnfrsf12*	*-*	up	HS vs. HT
*-*	*Tnfrsf14*	up	HS vs. HT

## Supplementary Materials

The following are available online at https://www.mdpi.com/2076-2615/10/9/1483/s1, Table S1. Sequence statistics for individual paired-end reads from RNA-Seq libraries of bighead carp; Table S2. Length distribution of the assembled transcriptomic contigs in bighead carp under hypoxia stress; Figure S1. Information for the decrease of DO and the number of loss-balanced fish with time; Figure S2. Length distribution of the unigenes from heart transcriptomes of bighead carp under hypoxia stress; Figure S3. Gene ontology classification of all unigenes in heart transcriptomes of bighead carp under hypoxia stress; Figure S4. Gene ontology classification of the DEGs from NC vs. HS groups of transcriptomes of bighead carp under hypoxia stress; Figure S5. Gene ontology classification of the DEGs from NC vs. HT groups of transcriptomes of bighead carp under hypoxia stress; Figure S6. KEGG pathway enrichment of the DEGs from NC and HS groups of transcriptomes of bighead carp under hypoxia stress; Figure S7. KEGG pathway enrichment of the DEGs from NC and HT groups of transcriptomes of bighead carp under hypoxia stress; Figure S8. Illustration of the qRT-PCR confirmation results for 9 DEGs from heart transcriptome of bighead carp under hypoxia stress. Gene expression levels are expressed as mean normalized ratios (*n* = 3, mean ± SE).
