# Supplementary material for: Cardiac Transcriptomics Reveals That MAPK Pathway Plays an Important Role in Hypoxia Tolerance in Bighead Carp (Hypophthalmichthys nobilis)
Source: Animals (Basel). 2020 Aug 24;10(9):1483. doi: 10.3390/ani10091483 (PMC7552209; doi:10.3390/ani10091483)
Supplement: Supplementary file 1 [file animals-10-01483-s001.pdf]

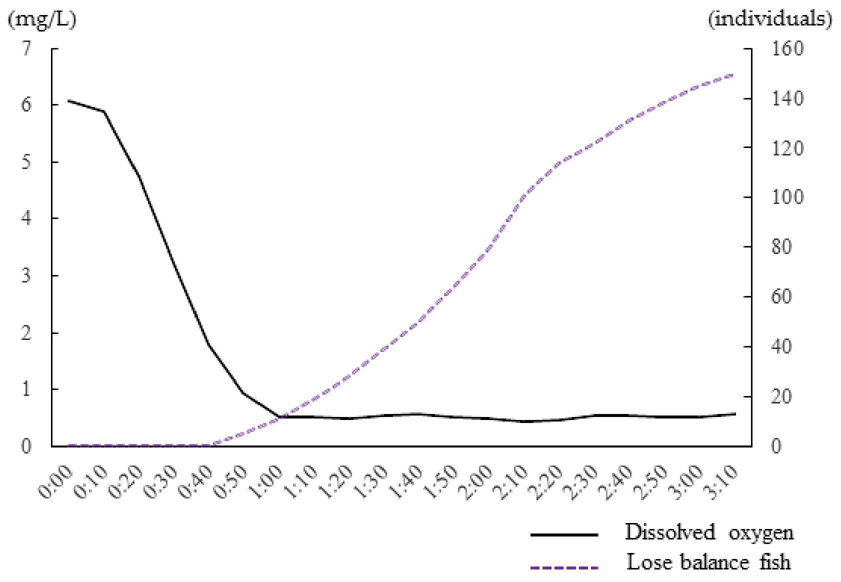

Figure S1. The information of decrease of DO and the number of loss balance fish with time.

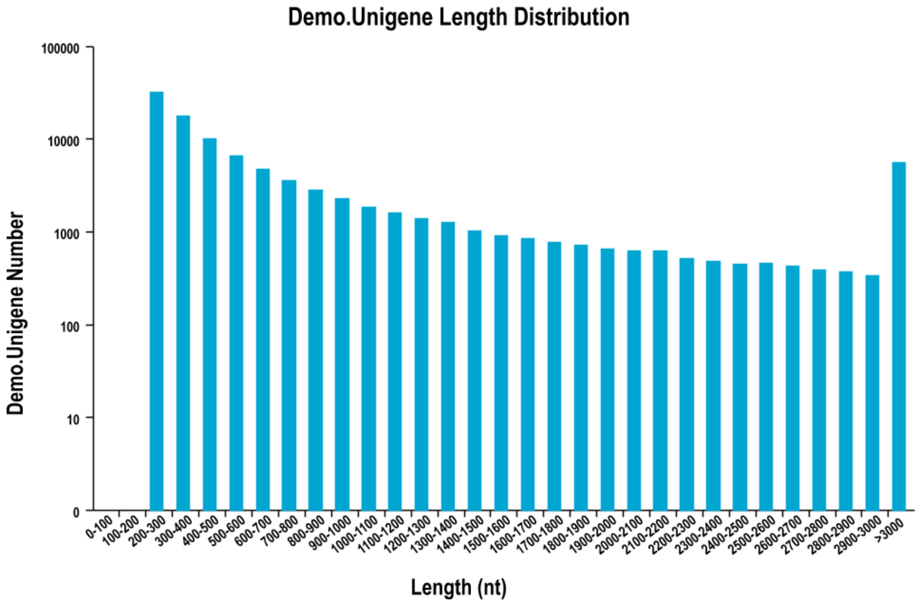

Figure S2 Length distribution of the unigenes from heart transcriptomes of bighead carp under hypoxia stress.

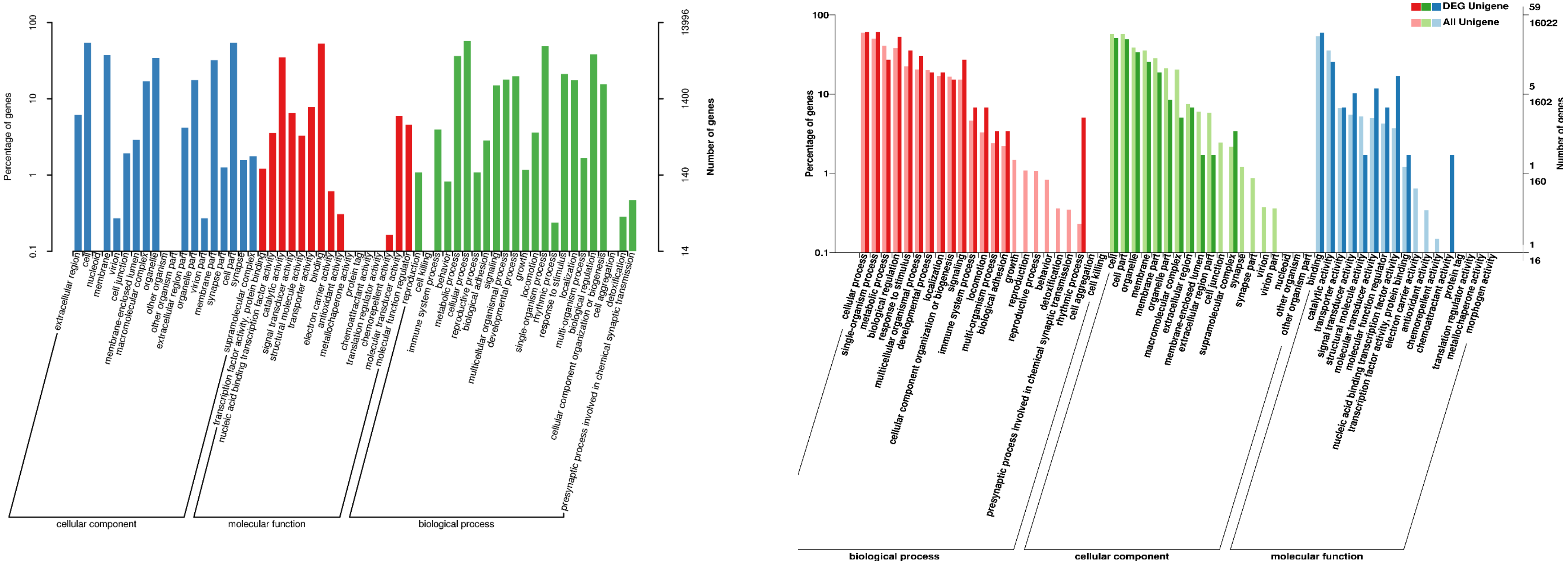

(**a**) (**b**)

Figure S3. (**a**). Gene ontology classification of all unigenes in heart transcriptomes of bighead carp under hypoxia stress; (**b**). Gene ontology classification of the DEGs from NC vs HS group of transcriptomes of bighead carp under hypoxia stress.

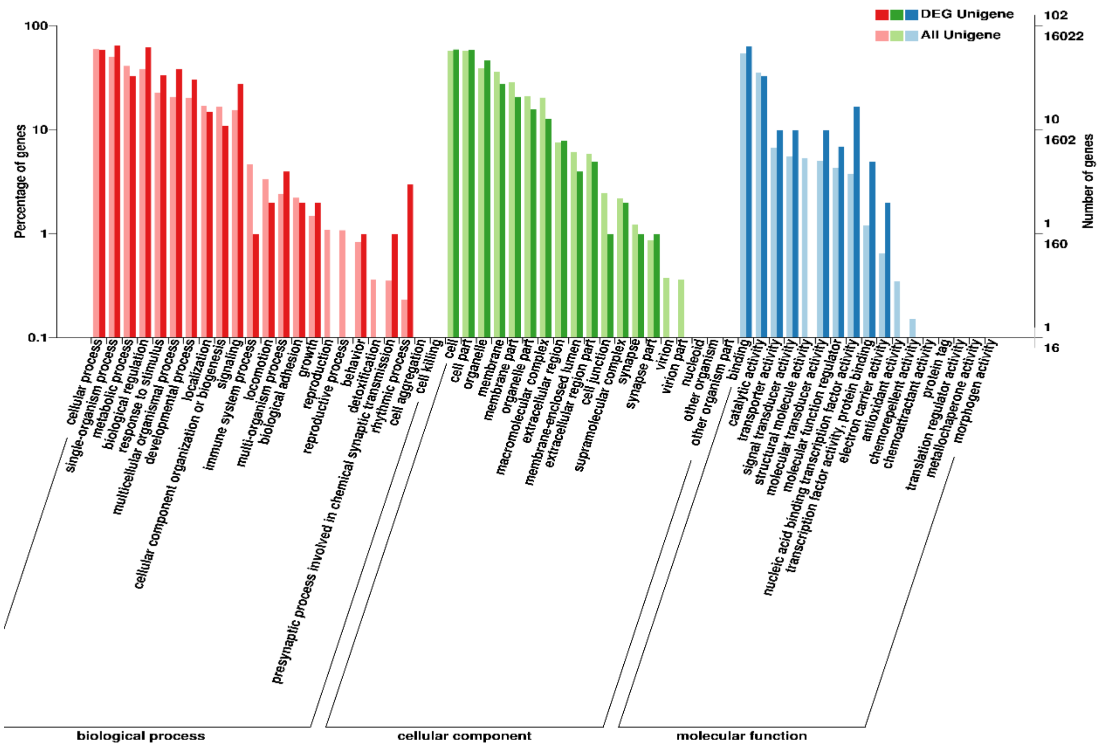

Figure S4. Gene ontology classification of the DEGs from NC vs HT group of transcriptomes of bighead carp under hypoxia stress.

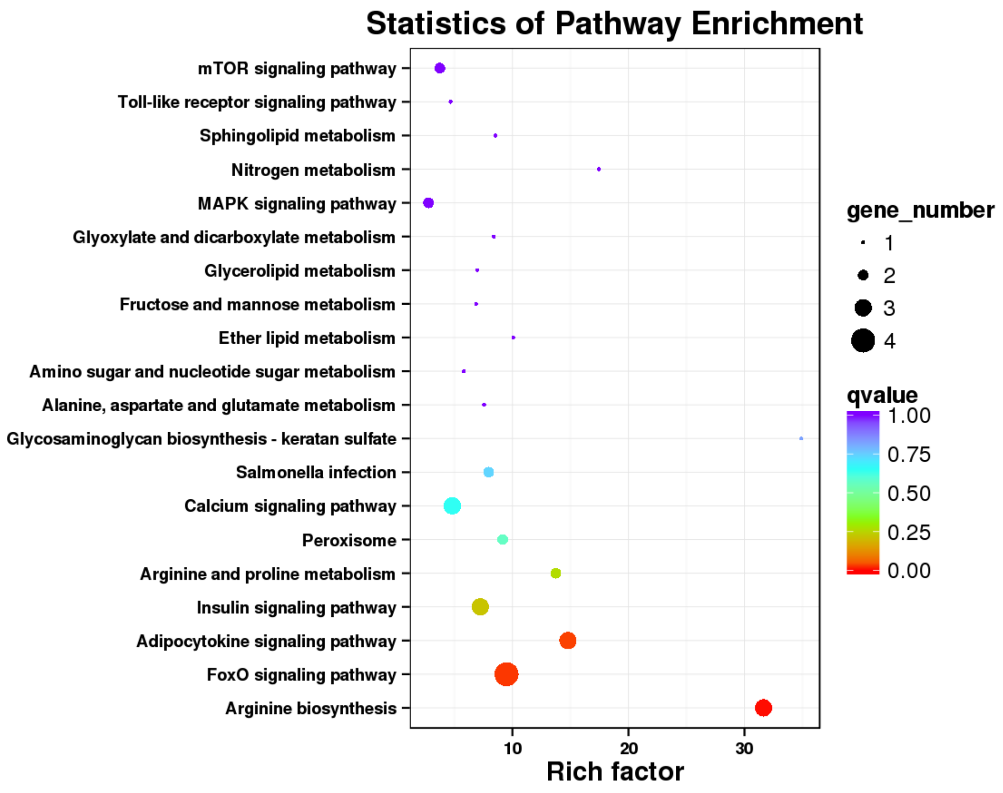

Figure S5. KEGG pathway enrichment of the DEGs from NC and HS groups of transcriptomes of bighead carp under hypoxia stress.

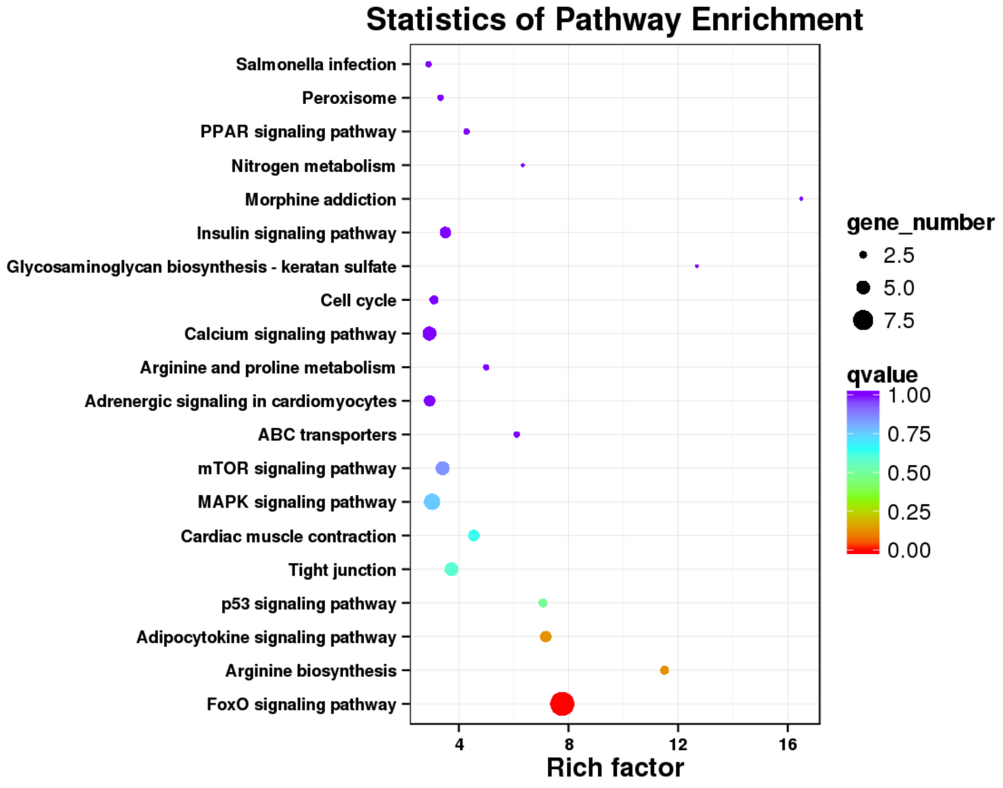

Figure S6. KEGG pathway enrichment of DEGs from NC and HT group of transcriptomes of bighead carp under hypoxia stress.

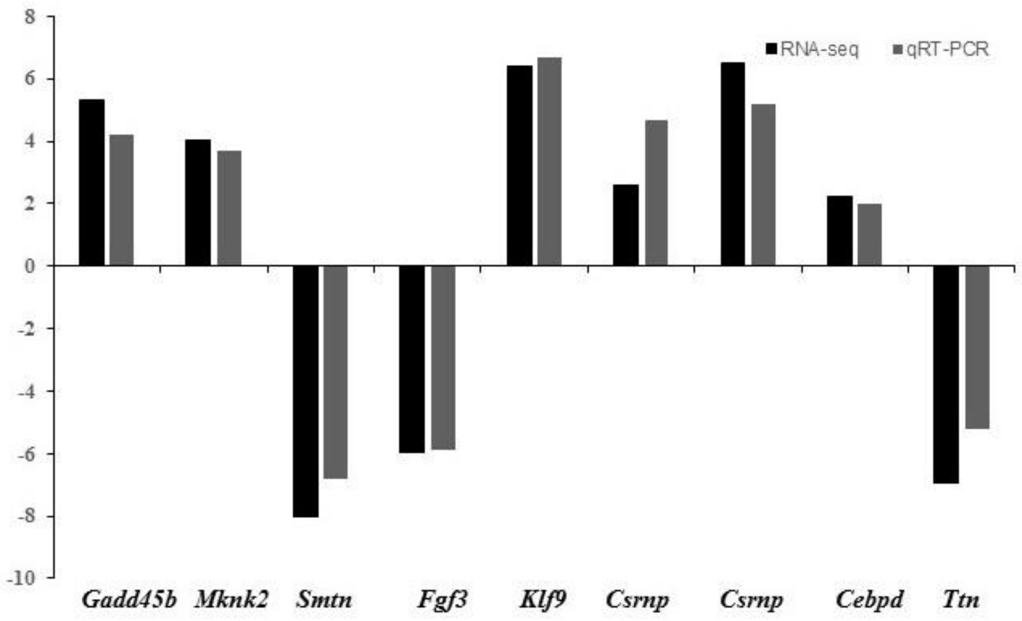

Figure S7. Illustration of qRT-PCR confirmation results for 9 randomly selected DEGs from the heart transcriptome of bighead carp under hypoxia stress. Gene expression levels are expressed as mean normalized ratios (n = 3, ± SE). β-Actin is used as a reference gene.

Table S1. Sequence statistics for individual paired-end reads from RNA-Seq libraries of bighead carp.

Samples	Clean reads	Q30	GC content	Mapped reads	Mapped ratio
HS1	27,118,672	95.07%	46.31%	22,426,752	82.70%
HS2	30,586,580	95.27%	46.41%	25,140,568	82.19%
HS3	29,372,299	95.09%	46.91%	23,745,084	80.84%
HT2	24,517,927	95.14%	46.77%	20,250,040	82.59%
HT2	21,941,179	93.96%	44.75%	18,204,812	82.97%
HT3	23,433,598	94.96%	46.07%	19,428,720	82.91%
HC1	31,345,416	95.17%	46.93%	25,874,293	82.55%
HC2	21,088,217	94.81%	44.72%	17,567,178	83.30%
HC3	23,534,816	95.03%	46.62%	19,298,044	82.00%

Table S2. Length distribution of the assembled transcriptomic contigs in bighead carp under hypoxia stress.

Length (bp)	Total Number of Unigenes	Percentage of Unigenes	Total Number of Transcripts	Percentage of Transcripts
200-300	31790	31.53%	36702	17.73%
300-500	27816	27.59%	37161	17.95%
500-1000	19960	19.80%	40266	19.45%
1000-2000	11009	10.92%	41885	20.24%
2000+	10240	10.16%	50952	24.62%
Total Number	100817		206970	
Total Length	85734049		303781753	
N50 Length	1633		2675	
Mean Length	850.392781		1467.757419	

© 2019 by the authors. Licensee MDPI, Basel, Switzerland. This article is an open access article distributed under the terms and conditions of the Creative Commons Attribution (CC BY) license (http://creativecommons.org/licenses/by/4.0/).
